# Epitope‐Resolved Digital SERS Profiling of Structurally Dynamic Antigens via a Multi‐Epitope Bispecific Antibody Framework

**DOI:** 10.1002/advs.75828

**Published:** 2026-05-27

**Authors:** Jing Wang, Quan Zhou, Kym Lowry, Christopher B. Howard, Matt Trau

**Affiliations:** ^1^ Key Laboratory of OptoElectronic Science and Technology for Medicine of Ministry of Education Fujian Provincial Key Laboratory of Photonics Technology Fujian Normal University Fuzhou China; ^2^ Australian Institute for Bioengineering and Nanotechnology (AIBN) The University of Queensland Brisbane QLD Australia; ^3^ Frazer Institute Faculty of Health, Medicine, and Behavioural Sciences The University of Queensland Royal Brisbane and Women's Hospital Brisbane QLD Australia; ^4^ Queensland Paediatric Infectious Diseases (QPID) Sakzewski Laboratory Queensland Children's Hospital Brisbane QLD Australia; ^5^ School of Chemistry and Molecular Biosciences The University of Queensland Brisbane QLD Australia

**Keywords:** bispecific antibody fragments, epitope‐resolved sensing, multi‐epitope immunoprofiling, mutation‐sensitive antigen detection, viral protein variants

## Abstract

Digital surface‐enhanced Raman scattering (SERS) immunoassays digitize epitope binding events to achieve ultrasensitive protein detection. However, existing implementations predominantly rely on single‐epitope recognition, yielding 1D molecular view of antigen structure and obscuring how conformational heterogeneity or mutation‐induced changes affect epitopes. This limitation cannot be resolved simply by combining multiple monoclonal antibodies, as heterogeneous conjugation and uncontrolled binding collapse epitope‐specific responses into pooled, non‐assignable signals. Here, we introduce EpiCount‐SERS (Epitope‐Resolved Digital Counting by SERS), a multi‐epitope digital SERS framework that enables epitope‐resolved immunochemical profiling. The platform employs nanobody‐based bispecific antibody fragments that pair epitope‐specific nanobodies with a unified anti‐methoxy polyethylene glycol conjugation domain, enabling orientation‐controlled attachment to spectrally encoded SERS nanotags. Epitope‐specific binding events are discretized into independent digital channels, allowing distinct epitopes on the SARS‐CoV‐2 receptor‐binding domain to be interrogated in parallel. Digital enumeration across these channels generates epitope‐resolved molecular fingerprints that capture information beyond single‐epitope assays. EpiCount‐SERS achieves sub‐ng mL^−^^1^ sensitivity for recombinant protein, detects inactivated virus at approximately 10^2^ copies µL^−^^1^, and classifies clinical nasopharyngeal swab samples with accuracy of 88.3% (area under the curve = 0.9467). Because epitope binders can be exchanged without altering the unified conjugation strategy, EpiCount‐SERS provides a scalable framework for digital immunochemical profiling of structurally dynamic protein targets.

## Graphical abstract

A multi‐epitope digital surface‐enhanced Raman spectroscopy framework enables epitope‐resolved immunoprofiling by integrating bispecific antibody fragments with spectrally encoded nanotags. This approach decouples binding events into independent digital channels, allowing structure‐aware antigen analysis. The platform achieves mutation‐discriminative detection and improves classification performance, offering a generalizable strategy for probing conformationally dynamic protein targets.

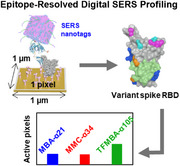

## 1 Introduction

Surface‐enhanced Raman scattering (SERS) provides a sensitive optical readout of molecular recognition events occurring on plasmonic nanoparticles [1, 2]. Recent advances in single‐particle SERS have enabled these recognition events to be interpreted digitally, giving rise to digital SERS immunoassays that convert nanoparticle binding into binary events [3, 4, 5]. This event‐based readout improves robustness over conventional intensity measurements and has expanded the SERS utility for highly sensitive immunochemical detection [6, 7]. Despite these advances, including prior implementations from our group, current digital SERS assays rely almost exclusively on single‐epitope recognition, offering only a 1D molecular perspective of the target antigen [6, 7, 8, 9]. This limitation restricts their ability to capture structural and conformational heterogeneity, as well as mutation‐induced variations in dynamic or evolutionarily variable protein antigens. As a result, digital SERS has not yet achieved epitope‐level resolution, nor has it been used to probe how changes in epitope accessibility influence molecular binding.

Although multiple monoclonal antibodies could, in principle, be used to target different epitopes, full‐length IgG architectures are fundamentally incompatible with epitope‐resolved digital detection. Their heterogeneous conjugation, variable nanoparticle orientation, and bivalent binding collapse epitope‐specific responses into pooled signals that cannot be unambiguously assigned to individual epitopes [10, 11, 12]. These molecular constraints prevent conventional antibody systems from providing the independent, structurally interpretable readouts required for multi‐epitope digital SERS. More importantly, this limitation is not due to the lack of suitable sensing components, but arises from the absence of a framework that can encode epitope‐specific binding events as independent and analyzable signals.

Bispecific antibody fragments (BsAbs) offer new opportunities for constructing well‐defined nanoparticle interfaces due to their compact size and programmable domain architecture [13, 14, 15]. However, existing BsAb‐based SERS assays, including our previous studies, have thus far been used only to improve orientation control for a single binding interaction and have not been configured to provide multiple, spectrally independent digital readouts [16]. In other words, the potential of BsAbs to enable parallel epitope‐specific channels has not yet been realized, leaving digital SERS fundamentally constrained to single‐epitope sensing. As a result, no current framework allows digital SERS to analyze how multiple epitopes on the same antigen contribute complementary structural information or to assess how mutations differentially perturb those epitopes. Critically, epitope‐resolved profiling does not arise from simply using multiple epitope‐specific probes, but from converting each epitope‐specific binding event into an independent digital information channel that can be analyzed and recombined.

Here, we introduce EpiCount‐SERS (Epitope‐Resolved Digital Counting by SERS), a multi‐epitope digital SERS framework designed to achieve epitope‐resolved immunochemical analysis. The platform integrates a panel of BsAb fragments directed toward spatially distinct antigenic epitopes with a unified nanoconjugation strategy, enabling consistent probe architecture while preserving epitope specificity. By digitally quantifying molecular recognition events across multiple epitopes, EpiCount‐SERS provides a structural perspective that is not accessible to single‐epitope assays. This is enabled by encoding binding events into independent epitope‐specific channels, allowing resolution at the level of individual epitopes. Such channel‐resolved representation provides the basis for interrogating how antigen structure influences molecular recognition and suggests broader potential for analyzing conformationally diverse or evolutionarily dynamic protein targets.

## 2 Materials and Methods

### 2.1 Clinical Samples

Clinical samples were provided by the Molecular Diagnostics Unit, Pathology Queensland. The study was conducted under approval by the Children's Health Queensland Hospital and Health Service Human Ethics Committee (HREC/22/QCHQ/85249), including a consent waiver, and ratified through the University of Queensland (2022/HE001497). Clinical samples were nasopharyngeal swabs collected in viral transport medium or phosphate buffer saline (PBS). Healthy control samples (n = 30) negative for SARS‐CoV‐2 were obtained and tested by reverse transcription‐quantitative polymerase chain reaction (RT‐qPCR) at the Molecular Diagnostics Unit, Pathology Queensland. Additional patient samples (n = 30) were provided by SpeeDx Pty Ltd (Australia). Samples lacking prior RT‐qPCR data underwent supplementary testing using an established assay to obtain Ct values [17]. All clinical testing was performed at the University of Queensland's Frazer Institute ‐ Herston.

### 2.2 BsAb Bioengineering

Sequences for BsAbs (α21, α34, α105), as reported previously [18, 19], were codon‐optimized for expression in Chinese Hamster Ovary (CHO) cells, and synthesized by Integrated DNA Technologies. The sequences were cloned into pcDNA3.1(+) vectors, incorporating an N‐terminal 6×His tag and a C‐terminal C‐Myc epitope tag. Transfection of 200 µg plasmids into ExpiCHO cells (6 × 10^6^ cells/mL, 200 mL) was performed using ExpiFectamine (ThermoFisher Scientific), following the manufacturer's protocol. After five days, secreted BsAbs were harvested from cell supernatants by centrifugation (400 × g, 10 min), filtered (0.2 µm PES), purified via HisTrap‐excel affinity chromatography (Cytiva), and buffer‐exchanged into PBS using a HiPrep 26/10 column (Cytiva).

### 2.3 Gel Staining

A total of 5.2 µL of BsAbs (∼1 mg/mL) was mixed with 2 µL of 4× NuPAGE LDS Sample Buffer and 0.8 µL of 10× Sample Reducing Agent (Thermo Fisher Scientific). The mixture, along with 8 µL of protein ladder (Bio‐Rad, 1610374), was denatured at 95°C for 5 min. Samples were then subjected to sodium dodecyl sulfate‐polyacrylamide gel electrophoresis (SDS‐PAGE) using 4%–12% NuPAGE Bis‐Tris gels, run at 200 V for 30 min in MES buffer. Following electrophoresis, the gel was rinsed with water and stained with SimplyBlue Safe Stain (ThermoFisher Scientific, LC6065).

### 2.4 Bio‐layer Interferometry (BLI)

The binding affinity (*K*_D_) of BsAb was assessed using an OctetRED system (ForteBio). Amino Propyl Silane (APS) biosensors were hydrated, immobilized with SARS‐CoV‐2 receptor‐binding domain (RBD) (100 µg/mL, in‐house expressed), and blocked with 1 mg/mL bovine serum albumin (BSA, Sigma). BsAb binding was measured at concentrations of 1.56‐100 nm. Kinetic parameters were analyzed using a 1:1 binding model (ForteBio software). In this analysis, the dissociation rate constant (*k_off_*) was obtained directly from the dissociation phase, while the association rate constant (*k_on_*) was derived from the association kinetics according to the relation *k_obs_* = *k_on_* ·*C* + *k_off_*, where *k_obs_* is the observed association rate constant at a given analyte concentration (*C*). The equilibrium dissociation constant was then calculated as *K_D_* = *k_off_/k_on_*, enabling accurate affinity determination even when equilibrium binding data alone were limited.

### 2.5 SERS Nanotag Preparation

Gold–silver alloy nanoboxes (NBs) were synthesized according to a previously published method [3]. The synthesized NBs were then functionalized by incubating with 2 µL of 1 mm Raman reporters, including 4‐mercaptobenzoic acid (MBA), 2,7‐mercapto‐4‐methylcoumarin (MMC), or 2,3,5,6‐tetrafluoro‐MBA (TFMBA), along with 2 µL of 1 mm thiol‐terminated methoxy polyethylene glycol (mPEG‐SH, Nanocs). After incubation for 1 h, excess reagents were removed by centrifugation, and the particles were resuspended in 200 µL of BsAbs (2.5 µg/mL) for 0.5 h. Following a second centrifugation step (600 × g, 8 min) to remove unbound BsAbs, particles were resuspended in 1% (wt.) BSA solution for blocking. Upon excitation, the nanotags produce distinct Raman signals at 1075, 1175, and 1375 cm^−1^, corresponding to MBA‐α21, MMC‐α34, and TFMBA‐α105, respectively.

### 2.6 Electron Microscopy Analysis

Transmission electron microscopy (TEM) and energy dispersive spectroscopy (EDS) were obtained using a JEM‐F200 operated at 200 kV.

### 2.7 Differential Centrifugal Sedimentation (DCS)

DCS (CPS DC24000 UHR) was conducted using a 14.4 mL sucrose gradient (8%–24% w/v) rotating at 24 000 rpm. Gradient parameters (density: 1.069 g/mL, refractive index: 1.36, viscosity: 1.505 cP) were calculated by manufacturer's specifications. Calibration preceded each run.

### 2.8 Nanoparticle Tracking Analysis (NTA)

Particle size distributions were measured using NanoSight N300 (Malvern Panalytical). For each sample, three sequential 60‐s videos were recorded and analyzed using NTA 3.4 software, with the camera level set at 11 and detection threshold set at 12.

### 2.9 Nanoflow Cytometry

BsAb‐functionalized NBs were incubated with recombinant RBD‐green fluorescent protein (GFP, molar ratio 1:1000). Samples were analyzed on a nanoFCM instrument (488 nm laser), with data acquisition via NF Profession 1.17 and FlowJo v10.7.1 software.

### 2.10 Chip Fabrication and Functionalization

Chips incorporating asymmetric gold electrode arrays on glass substrates and polydimethylsiloxane (PDMS) wells were fabricated via standard photolithography and lift‐off processes [20, 21]. Briefly, electrode structures consisting of an asymmetric inner circular electrode (diameter = 1000 µm) and an outer ring‐shaped electrode (width = 120 µm, spacing = 1000 µm) were defined on 4‐inch. Borofloat glass wafers using a direct laser‐written chrome mask, followed by photoresist coating, UV exposure, and development. Titanium (10 nm) and gold (200 nm) layers were subsequently deposited by electron‐beam evaporation, followed by overnight lift‐off. PDMS layers were prepared by curing elastomer solution (Sylgard 184, Dow) at 80°C for 20 min, and microwells with 6 mm diameter and 5 mm depth were punched to serve as sample chambers. The PDMS layer was thermally bonded to the electrode‐patterned glass substrate overnight at 65°C. The resulting gold‐patterned electrodes generate nanoscale mixing under an applied alternating current electric field, thereby enhancing molecular interaction efficiency [22].

### 2.11 Digital SERS Immunoassays

Gold electrodes were functionalized by incubating with 20 µL of 5 mm dithiobis(succinimidyl propionate) (DSP, Thermo Fisher Scientific) for 1.5 h at room temperature, rinsed once with ethanol and three times with PBS, and subsequently coated with CR3022 monoclonal antibody (5 µL, 10 µg/mL, Sino Biological) for 1.5 h. After a PBS wash, the surfaces were blocked with 1% (w/v) BSA overnight at 4°C.

For immunoassays, 50 µL aliquots of samples were pipetted into each microwell and incubated under an alternating current electric field (800 mV, 500 Hz) for 30 min to facilitate efficient antigen capture. After three PBS washes, 25 µL of BsAb‐functionalized SERS nanotags were added and incubated under identical nanomixing conditions for 45 min, followed by another three PBS washes.

Raman mapping was conducted on a WITec alpha300 R microspectrometer using 632 nm laser excitation, a 100× objective (NA = 0.9), and a spectral resolution of ∼1 cm^−1^. Data were acquired with 0.01 s integration time per spectrum (per pixel), and a 60 × 60 µm mapping area with 1 µm step size. False‐color Raman images were generated using WITec Suite FIVE software.

Active pixels were defined as those with a signal‐to‐noise ratio (S/N) greater than 3, where the signal corresponds to the peak intensity and the noise is estimated from the local spectral baseline. Pixels with S/N ≤ 3 were classified as inactive. The resulting binary events were digitally counted to generate the final readout. This digital SERS binarization strategy can offer up to an 11‐fold enhancement compared with conventional intensity‐based analysis [6].

### 2.12 Statistical Analysis

Receiver operating characteristic (ROC) analysis, unpaired t test (two‐tailed), two‐way ANOVA, multiple logistic regression analysis (MLR), and heatmap generation were performed using GraphPad Prism 10.1.2. A fivefold cross‐validation of the MLR model was performed using Orange (version 3.34).

## 3 Results and Discussion

### 3.1 Conceptual Framework of EpiCount‐SERS for Multi‐Epitope Digital Immunoprofiling

**FIGURE 1:**
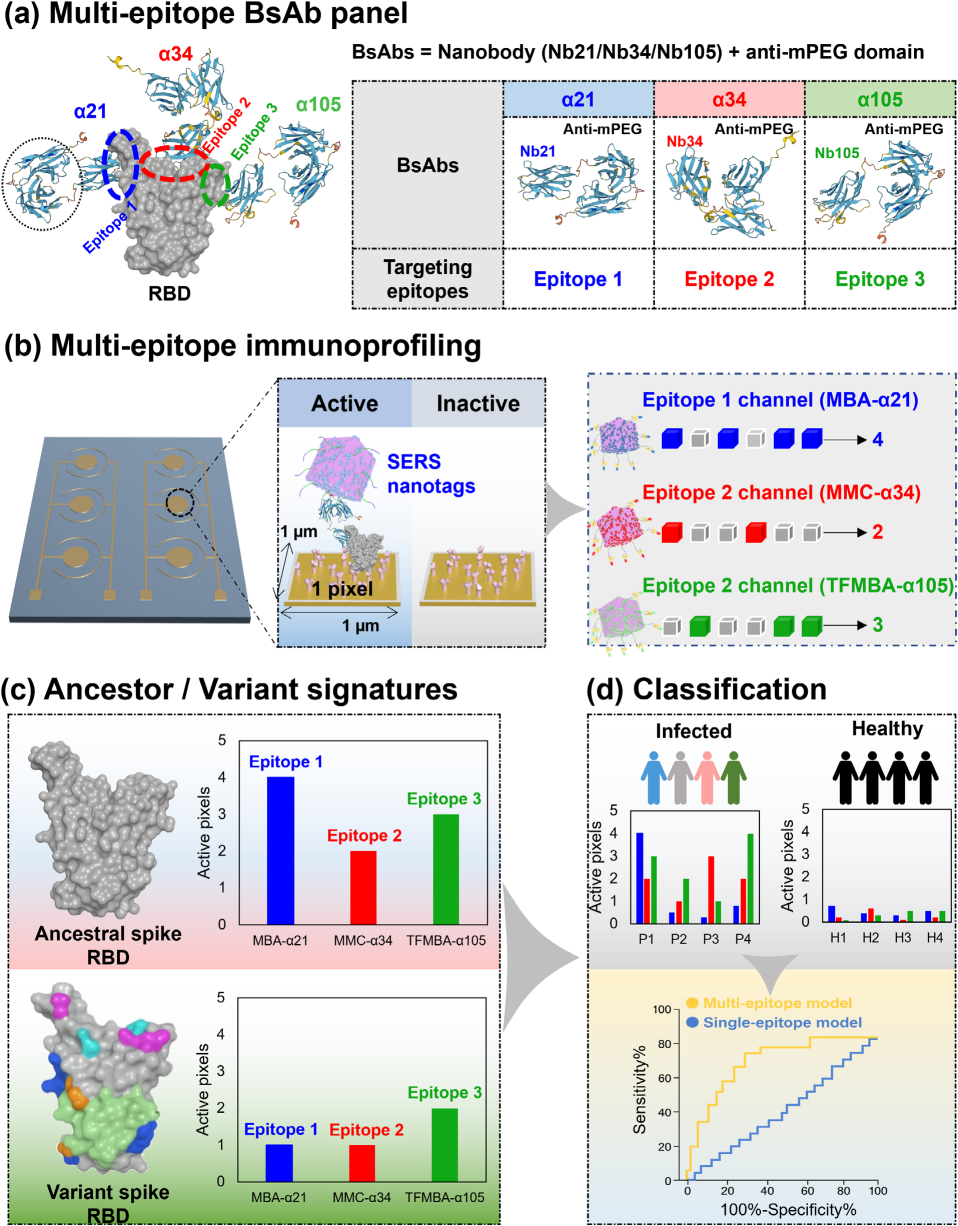
Conceptual framework of EpiCount‐SERS for multi‐epitope, epitope‐resolved digital immunoprofiling. (a) Three BsAbs (α21, α34, and α105) recognizing non‐overlapping epitopes within the SARS‐CoV‐2 RBD constitute a multi‐epitope molecular interrogation panel. (b) Raman mapping converts epitope‐specific binding events of spectrally encoded SERS nanotags into digital “active pixel” signals. (c) Multi‐epitope digital readout generates distinct channel‐resolved signal patterns in response to RBD variants with altered epitope accessibility. (d) Integration of multiple epitope‐resolved digital channels enables downstream classification analysis based on combined molecular signatures.

EpiCount‐SERS integrates multi‐epitope molecular recognition with single‐particle activity‐based SERS readout to enable epitope‐resolved immunoprofiling. A panel of BsAb‐functionalized SERS nanotags is constructed, in which each BsAb (α21, α34, α105) selectively recognizes a distinct epitope within the RBD of spike proteins. These BsAbs are engineered by fusing epitope‐specific nanobodies (Nb21, Nb34, or Nb105) to a unified anti‐mPEG anchoring domain (Figure 1a), enabling orientation‐controlled conjugation to mPEG‐grafted SERS nanotags. The resulting nanotags (MBA‐α21, MMC‐α34, and TFMBA‐α105) incorporate distinct Raman reporters (MBA, MMC, and TFMBA), encoding epitope‐specific binding into separate detection channels (Figure 1b).

Raman mapping identifies reporter signals as localized “active pixels”, which are binarized into digital events without requiring a strict one‐to‐one correspondence with individual nanotags or targets (Figure 1b). Due to the diffraction‐limited spatial resolution of confocal Raman microscopy, multiple nanotags may contribute to a single pixel; however, measurements are performed under a low‐occupancy regime, where the mean occupancy per pixel remains low (λ ∼ active pixels/total pixels, e.g., λ ∼ 0.19 at the highest signal level), and the probability of multiple events per pixel is correspondingly minimal (following Poisson statistics, *P* (k ≥ 2) = 1−(1 + λ) × e^−λ^, which is ∼1.6% at λ = 0.19). Under these conditions, active pixel counts provide a reliable quantitative measure of binding events. Importantly, any deviation from single‐event occupancy scales with analyte concentration and does not distort quantification, preserving an approximately linear relationship between digital counts and analyte level within the working range.

These channel‐resolved digital events enable parallel quantification of epitope‐specific binding, where differences across channels reflect variations in epitope accessibility. Their integration yields multi‐epitope digital signatures encoding complementary structural information for the RBD of ancestral and variant spike proteins, where mutations predominantly occur (Figure 1c). When applied to clinical samples, this framework supports classification with improved sensitivity over single‐epitope assays by preserving, rather than collapsing, epitope‐specific information (Figure 1d).

### 3.2 Optimizing BsAb‐SERS Nanotags for Orientation‐Controlled, Multi‐Epitope Recognition

**FIGURE 2:**
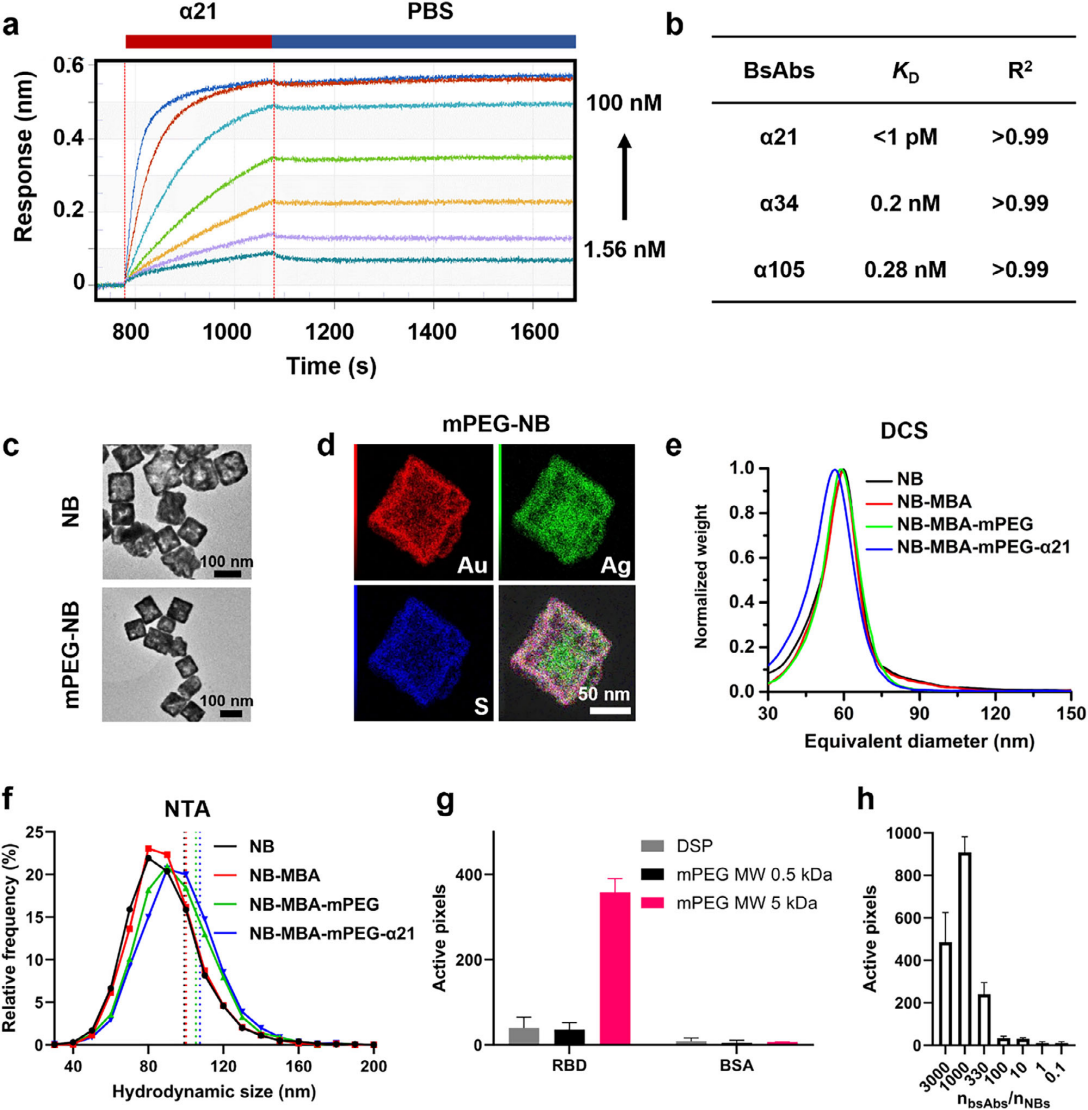
Assembly and characterization of BsAb‐functionalized SERS nanotags for multi‐epitope digital sensing. (a,b) BLI measurements illustrating the binding interactions between BsAb (α21, α34, α105) and their corresponding targets. (c,d) TEM images and EDS mapping of mPEG‐modified NBs. (e,f) DCS and NTA used to monitor size evolution during stepwise nanotag assembly. (g) EpiCount‐SERS digital responses of BsAb‐functionalized nanotags prepared using different surface linkers (DSP and mPEG of different lengths). (h) EpiCount‐SERS digital responses of BsAb‐functionalized nanotags prepared with different BsAb‐to‐NB molar ratios. Error bars in Figure 2g,h represent the standard deviation (SD) from three independent experiments.

We first assessed the molecular integrity and binding performance of the three BsAbs. SDS‐PAGE confirmed their expected molecular weights (∼41 kDa for α21 and ∼42 kDa for α34 and α105; Figure S1). BLI analysis across 1.56–100 nm BsAbs revealed *K*_D_ of <1 pM, 0.2 nm, and 0.28 nm for α21, α34, and α105, respectively (Figure 2a,b), consistent with previous reports [23]. These results verify successful synthesis and preserved high‐affinity binding of all BsAbs.

Gold‐silver alloy NBs were then characterized before and after mPEGylation. TEM imaging showed their hollow cubic morphology remained intact following mPEG grafting, while EDS mapping confirmed the presence of Au, Ag, and S signals—the latter arising from thiolated mPEG—indicating robust Au–S covalent anchoring (Figure 2c,d). The alloy NBs exhibited a SERS enhancement factor of ∼3.2 × 10^6^ (Figure S2), attributable to sharp edges and strong plasmonic coupling within the hollow interior that generate intense electromagnetic “hot spots” [3].

Because surface passivation critically influences both colloidal stability and Raman reporter loading, we optimized the density of mPEG on NBs. Increasing mPEG amounts (2–10 µL at 1 mm) progressively reduced equivalent diameters measured by DCS, reflecting decreased effective density and increased hydrophilicity from thicker coatings (Figure S3a). However, excessive mPEG significantly suppressed Raman reporter loading (Figure S3b). A loading of 2 µL mPEG was therefore selected as an optimal compromise between surface passivation and SERS performance.

BsAb conjugation onto reporter‐labeled NBs was confirmed by both DCS and NTA. DCS showed reduced equivalent diameters after α21 attachment (Figure 2e), while NTA revealed stepwise increases in hydrodynamic size from 99.2 ± 1.3 nm (NBs) to 107.5 ± 0.4 nm (NB–MBA–mPEG–α21), corresponding to an overall ΔDh of ∼8.4% relative to the bare NBs (Figure 2f). Together, these orthogonal characterizations confirm effective, layer‐by‐layer assembly of BsAbs onto the SERS nanotags.

To further validate the reproducibility of nanotag synthesis and the stability of Raman encoding, stepwise SERS spectra were obtained during surface modification (Figure S4a). The spectra correspond to the same nanotag before and after BsAb attachment, demonstrating that the characteristic Raman peaks are preserved throughout functionalization. In addition, the final spectra of all three nanotags (MBA‐α21, MMC‐α34, and TFMBA‐α105) exhibit distinct and non‐overlapping peaks (Figure S4b), confirming their suitability for multiplexed detection.

To verify functional integrity, α21‐, α34‐, and α105‐modified nanotags were incubated with GFP‐tagged RBDs and analyzed by nanoflow cytometry. Each nanotag maintained its intended epitope specificity, with labeling efficiencies of 9.8%, 59%, and 2.74%, respectively (Figure S5). Additional DCS experiments further established epitope selectivity: immunocomplexes formed readily with as little as 10 ng RBD for α21/α34‐modified NBs, whereas α21/α105 nanotags required ≥ 100 ng RBD (Figure S6). Increasing RBD concentrations yielded proportionally higher complex formation, confirming dose‐dependent binding and non‐overlapping epitope recognition [18].

Although the compact BsAb format reduces steric hindrance at the molecular recognition interface compared with conventional IgG‐based probes, the nanoscale SERS nanotag can still introduce residual spatial crowding during multi‐epitope interrogation. We therefore examined whether linker architecture modulates effective epitope accessibility in the EpiCount‐SERS assay. Conjugation of α21 using a 5 kDa mPEG linker resulted in substantially enhanced RBD‐associated digital signals, with ∼tenfold and ninefold increases compared to 0.5 kDa mPEG and DSP linkers, respectively (Figure 2g). This linker‐length dependence indicates that spacer architecture critically influences molecular engagement, with increased nanoscale spacing improving nanotag access to target epitopes.

BsAb loading was further evaluated across a wide α21‐to‐nanotag molar ratio range (0.1–3000). An intermediate ratio of 1000 yielded the highest digital response (Figure 2h), indicating a balance between probe surface density and accessible binding sites: low loading limits binding probability, whereas excessive surface coverage introduces crowding or partial masking at the nanotag interface.

Collectively, these results show that, despite the reduced steric hindrance afforded by nanobody‐based BsAbs, overall assay performance remains governed by nanotag architecture. Optimizing spacer length and probe surface coverage is therefore essential to minimize spatial interference and maximize effective epitope engagement. These findings further establish a framework for engineering BsAb‐functionalized SERS nanotags, providing a molecular basis for multi‐epitope recognition and epitope‐resolved profiling in the EpiCount‐SERS platform.

### 3.3 Analytical Performance of EpiCount‐SERS for RBD and Intact Virus Detection

**FIGURE 3:**
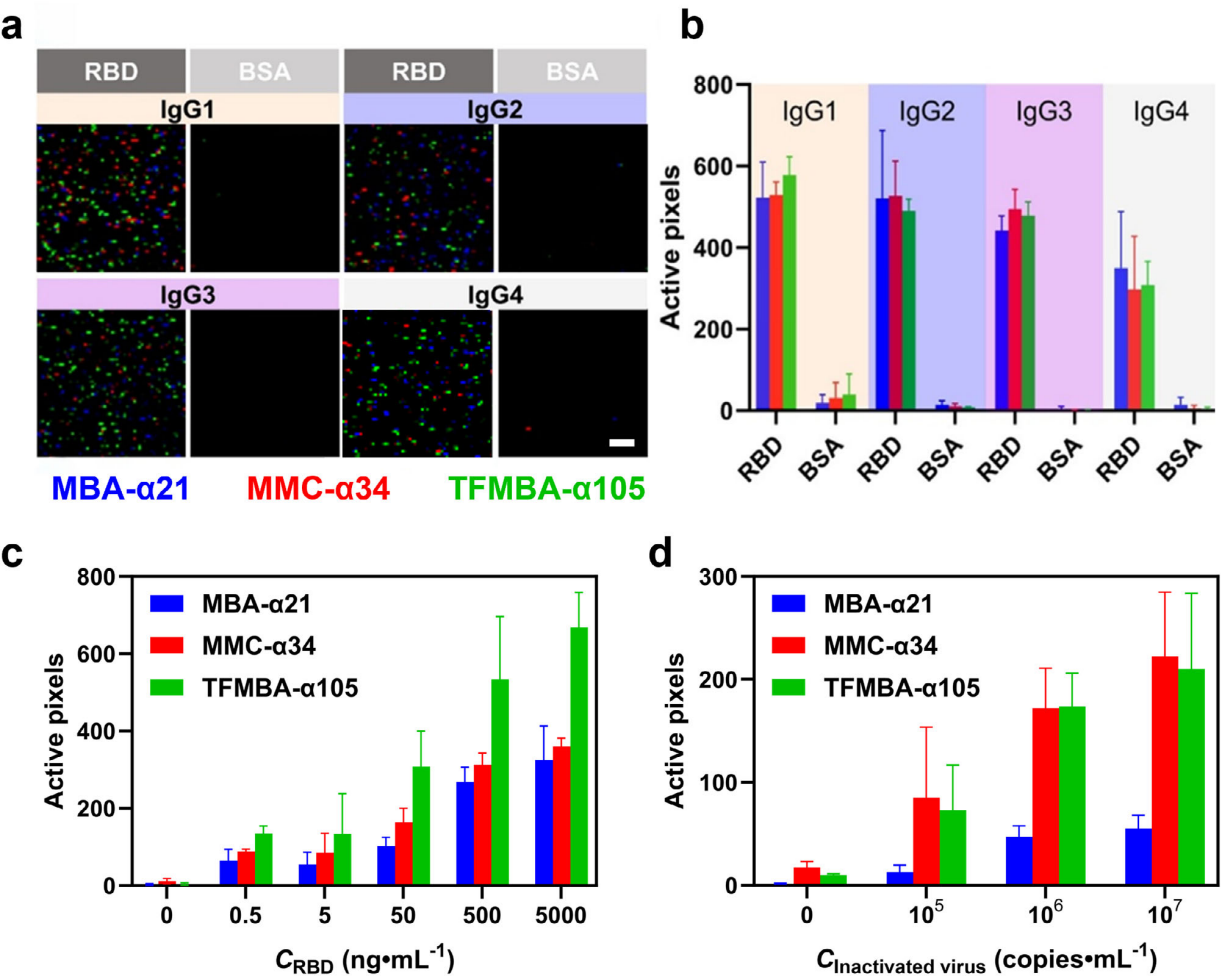
Analytical performance of EpiCount‐SERS for recombinant RBD and inactivated SARS‐CoV‐2 detection. (a) Representative false‐color Raman maps showing digital active‐pixel patterns obtained using different CR3022 IgG subclasses (IgG1–IgG4) for recombinant RBD capture, with the BSA as controls. (b) Quantification of active‐pixel counts corresponding to panel (a), comparing RBD and BSA across the four IgG subclasses. (c) Digital active‐pixel responses to increasing concentrations of recombinant RBD detected using the three epitope‐resolved SERS nanotags (MBA–α21, MMC–α34, and TFMBA–α105). (d) Digital active‐pixel responses to increasing concentrations of inactivated SARS‐CoV‐2 virus detected using the same nanotag panel. Data are presented as mean ± SD (n = 3 independent experiments). Scale bar, 10 µm.

Because EpiCount‐SERS uses surface‐immobilized CR3022 antibodies for antigen capture prior to multi‐epitope digital readout, we first optimized the capture antibody format to ensure structural compatibility with downstream epitope‐resolved sensing. CR3022 binds an epitope spatially distinct from those recognized by the three BsAbs [24, 25], which themselves target mutually non‐overlapping epitopes [18], thereby establishing a capture‐detection configuration that preserves accessibility of all three epitope‐specific channels. This spatial separation ensures that RBD capture is achieved without steric interference with epitope‐resolved digital interrogation. IgG subclasses are known to differ in Fc geometry, hinge flexibility, and lysine distribution, all of which can influence immobilization orientation and the presentation of RBD epitopes to BsAb‐functionalized SERS nanotags; we therefore systematically compared CR3022 IgG1–IgG4 to assess their suitability for multi‐epitope digital interrogation. A 5% BSA solution served as the negative control. All IgG subclasses enabled specific antigen capture compatible with downstream epitope‐resolved digital readout (Figure 3a,b); however, IgG4 exhibited reduced capture efficiency, consistent with its comparatively less favorable physicochemical characteristics [26]. Among the subclasses tested, IgG1 exhibited the most stable and reproducible digital response after storage for 7 days at 4°C (Figure S7) and was therefore selected as the optimized capture ligand for EpiCount‐SERS.

To evaluate the baseline digital sensitivity enabled by this capture–readout architecture, we performed a concentration‐dependent titration of recombinant RBD from 5 to 5000 ng/mL in PBS. As shown in Figure 3c, active‐pixel counts increased monotonically with antigen concentration and remained clearly detectable down to 0.5 ng/mL, demonstrating the high sensitivity of the digital SERS readout. Compared with conventional sandwich ELISA, our previous study using a similar BsAb‐enabled platform demonstrated an improvement in detection limit from 7.31 ng/mL (ELISA) to 3.97 ng/mL (digital SERS), corresponding to an approximately twofold enhancement [16]. In addition to this sensitivity improvement, the assay achieves substantially reduced reagent consumption, requiring approximately 20‐fold less anti‐RBD and ∼twofold less sample. Furthermore, it enables multiplexed epitope‐level detection within a single assay, which is not readily achievable using conventional ELISA formats.

A similar trend was observed for inactivated SARS‐CoV‐2 virus (Figure 3d), where active pixels were detectable at 100 copies/µL and scaled proportionally with viral load. These detection sensitivities are comparable to, or exceed, those reported for state‐of‐the‐art SERS‐based immunoassays [9, 27], indicating that distributing binding events across multiple epitope‐resolved digital channels preserves high analytical sensitivity.

Together, these experiments establish the high intrinsic sensitivity of EpiCount‐SERS for both recombinant protein and intact virus detection, providing a quantitative performance baseline for subsequent epitope‐resolved and mutation‐sensitive analyses. We therefore next examined whether multi‐epitope digital readout can distinguish structural perturbations across spike protein variants.

### 3.4 Epitope‐Resolved and Mutation‐Sensitive Digital Profiling of Spike Proteins

**FIGURE 4:**
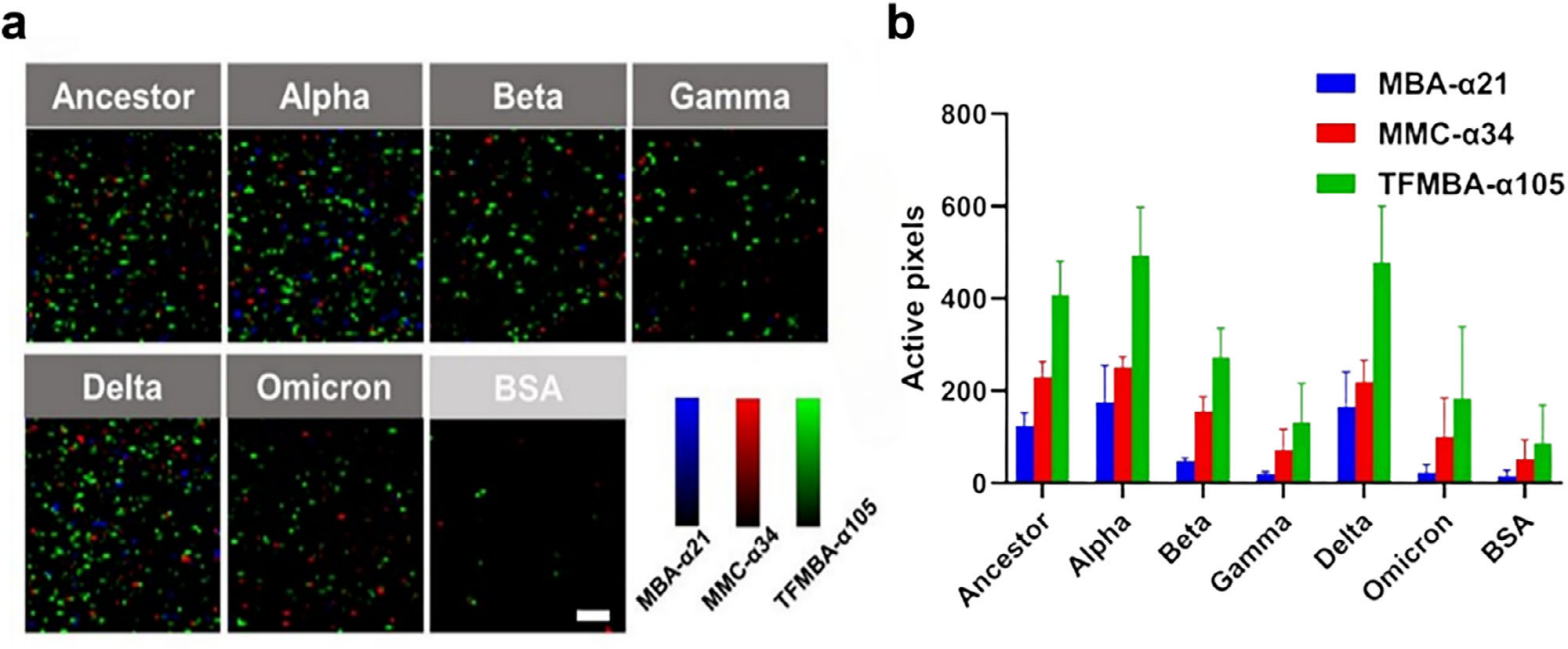
Epitope‐resolved digital profiling of SARS‐CoV‐2 spike protein variants using EpiCount‐SERS. (a) Representative false‐color Raman maps showing epitope‐resolved digital readouts generated by the three SERS nanotag channels (MBA–α21, MMC–α34, and TFMBA–α105) in response to spike proteins from the ancestral SARS‐CoV‐2 strain and multiple variants (Alpha, Beta, Gamma, Delta, and Omicron). BSA was included as a negative control. (b) Quantification of active‐pixel counts corresponding to panel (a) for each epitope‐specific nanotag channel across variants. Data are presented as mean ± SD (n = 3). Scale bar, 10 µm.

SARS‐CoV‐2 expresses a trimeric spike protein whose RBD accumulates frequent mutations that can alter epitope accessibility [28]. Building on the established analytical sensitivity of EpiCount‐SERS, we next asked whether its multi‐epitope digital readout can resolve mutation‐dependent perturbations in epitope accessibility across structurally divergent spike variants. To this end, we profiled recombinant spike variants using the MBA‐α21, MMC‐α34, and TFMBA‐α105 nanotags. Variants such as Beta, Gamma, and Omicron carry E484K/A substitutions that are known to perturb the epitope addressed by α21 [29, 30], and accordingly, the MBA‐α21 channel signals showed the most pronounced reductions across these variants. In contrast, MMC‐α34 and TFMBA‐α105 channels exhibited distinct, variant‐dependent response patterns—generally retaining detectable activity while displaying varying degrees of attenuation depending on the specific mutation context (Figure 4a,b). Importantly, these channel‐specific redistributions reflect differential epitope perturbation rather than uniform signal loss, demonstrating that epitope‐resolved digital readout can capture structural alterations that would be masked in single‐epitope or intensity‐averaged assays.

Importantly, the three epitope channels contribute independently and complementarily to the overall digital readout, enabling EpiCount‐SERS to remain robust against mutation‐induced perturbations affecting any single epitope. By preserving epitope identity at the level of individual digital binding events, the integrated multi‐channel readout maintains stable detection performance even when individual channels exhibit variant‐specific attenuation. Together, these results establish EpiCount‐SERS as a mutation‐discriminative, epitope‐resolved digital immunoprofiling framework, capable of decoding structural heterogeneity in dynamically evolving spike proteins.

Despite this robustness, the accuracy of epitope‐resolved digital profiling may still be influenced by factors such as epitope accessibility and conformational variability, differences in binding affinity among BsAbs, as well as S/N characteristics and thresholding criteria in digital event classification, which should be carefully considered and systematically evaluated in future studies.

### 3.5 Clinical Validation of Multi‐Epitope Digital Sensing Using EpiCount‐SERS

**FIGURE 5:**
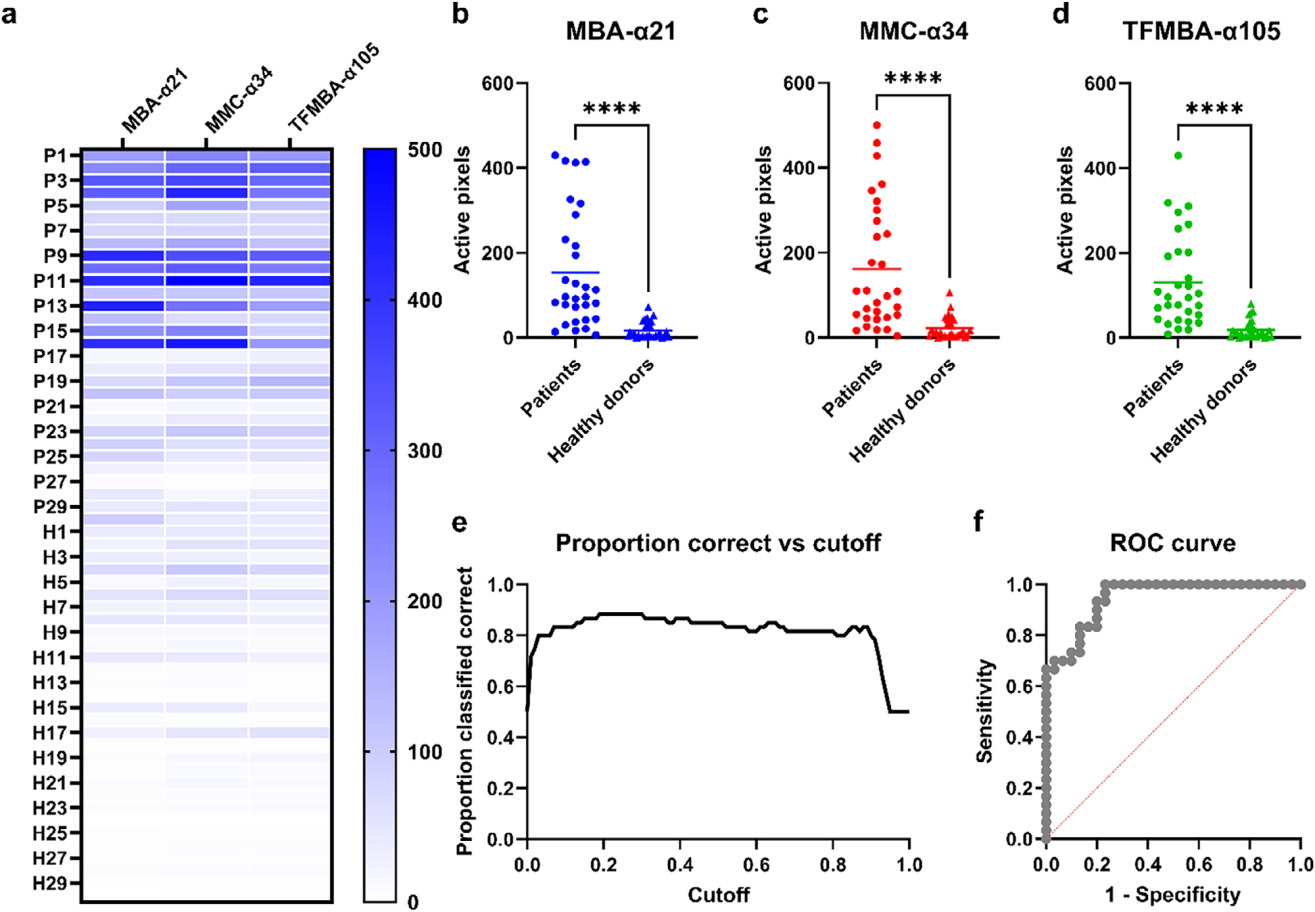
Multi‐epitope digital analysis of clinical samples using EpiCount‐SERS. (a) Heatmap visualization of digital signals from the three SERS nanotag channels across clinical samples and healthy controls. (b–d) Channel‐wise distributions of active‐pixel signals for MBA–α21, MMC–α34, and TFMBA–α105 nanotags, respectively. (e) Classification accuracy as a function of the decision cutoff applied to the model output. (f) ROC curve for the classification model. ^****^ *P *< 0.0001. Statistical significance was assessed using the unpaired two‐tailed t‐test.

We next evaluated the clinical performance of the EpiCount‐SERS using nasopharyngeal swab samples from a cohort comprising 26 individuals infected with the ancestral SARS‐CoV‐2 strain, 4 individuals infected with the Alpha variant, and 30 RT‐qPCR–confirmed healthy controls. A heatmap summarizing active‐pixel counts across the three BsAb‐defined epitope channels is shown in Figure 5a. As illustrated in Figure 5b–d, infected individuals exhibited markedly elevated active‐pixel counts relative to healthy controls across one or more channels, consistent with the epitope‐resolved digital signatures observed in recombinant antigen experiments (Figure 4). The differences between groups were further quantified using 95% confidence intervals (CI) and effect sizes. The 95% CI of the mean differences were −187.7 to −86.91 (MBA‐α21), −193.7 to −84.14 (MMC‐α34), and −152.5 to −71.55 (TFMBA‐α105), all excluding zero. The corresponding effect sizes (Cohen'S D) were 1.43, 1.33, and 1.46, respectively, indicating large effect sizes across all three channels. Together, these results quantitatively confirm strong group separation despite partial overlap in the distributions.

To quantify diagnostic performance, we first evaluated each epitope channel individually. Single‐epitope ROC analyses yielded AUCs of 0.9278, 0.8889, and 0.9217 for α21‐, α34‐, and α105‐nanotags, respectively (Figure S8), indicating that while each channel provides discriminative power, their performance varies depending on epitope sensitivity to viral mutations. We then applied MLR to integrate the three channels. The combined multi‐epitope model achieved a specificity of 90%, a sensitivity of 80%, and an overall accuracy of 85% at the default cutoff of 0.5, with accuracy increasing to 88.3% when the threshold was adjusted to 0.19–0.30. ROC analysis of the integrated classifier yielded an AUC of 0.9467, substantially outperforming the single‐epitope models. This performance gain reflects the core design principle of EpiCount‐SERS: complementary epitopes contribute non‐redundant molecular information, enabling robust classification even when the response of an individual epitope is attenuated by mutation‐associated changes. As a result, multi‐epitope digital immunoprofiling provides a more mutation‐discriminative and clinically robust diagnostic framework than traditional single‐epitope digital SERS assays.

To further evaluate model robustness and generalizability, a fivefold cross‐validation was performed. The cross‐validated model achieved an AUC of 0.917, with a sensitivity of 76.7% and specificity of 90.0% at a classification threshold of 0.5. The corresponding confusion matrix is shown in Figure S9. These results suggest stable classification performance across data splits, supporting the robustness and generalizability of the model.

It is also important to note that although the clinical cohort primarily comprised ancestral SARS‐CoV‐2 samples and a limited number of Alpha cases, the variant relevance of EpiCount‐SERS is established at the mechanistic level through epitope‐resolved digital analysis of recombinant spike proteins with defined mutations. The clinical data, therefore, validate assay feasibility and diagnostic performance in real samples, rather than aiming to provide exhaustive epidemiological coverage of circulating variants.

Beyond SARS‐CoV‐2, the design principles of EpiCount‐SERS are broadly applicable to structurally dynamic or mutation‐prone protein targets. Because the platform decouples nanoparticle conjugation chemistry from epitope specificity through a unified anti‐mPEG module, BsAbs recognizing additional epitopes, or entirely different protein systems, can be readily incorporated without re‐engineering the sensing architecture. This modularity positions EpiCount‐SERS as a generalizable framework for multi‐epitope, mutation‐discriminative immunochemical profiling across diverse biomedical contexts, extending its utility to a wide range of conformationally heterogeneous or evolutionarily variable biomolecules.

## 4 Conclusions

In conclusion, EpiCount‐SERS establishes a conceptual and technical advance in digital immunochemistry by enabling epitope‐resolved sensing through digital decomposition of epitope‐specific binding events, rather than parallel intensity measurements, using a multi‐epitope BsAb panel integrated with multiplexed Raman digitization. This multi‐channel molecular architecture overcomes the intrinsic limitations of single‐epitope assays, providing mutation‐discriminative, structurally informed antigen detection across both spike proteins and clinical specimens. Additionally, by coupling modular BsAb recognition, the unified conjugation strategy offers a scalable foundation for extending multi‐epitope digital profiling to other conformationally heterogeneous or evolutionarily dynamic protein targets. Collectively, this work expands digital SERS from 1D, intensity‐based molecular recognition toward a generalizable, structure‐aware analytical framework for next‐generation immunochemical analysis. Future work focusing on broader validation across additional target systems and diverse biological matrices will be important to further establish its general applicability.

## Conflicts of Interest

The authors declare no conflict of interest.

## Supporting information




**Supporting File**: advs75828‐sup‐0001‐SuppMat.docx.

## Data Availability

The data that support the findings of this study are available on request from the corresponding author. The data are not publicly available due to privacy or ethical restrictions.
