# Supplementary material for: Epitope‐Resolved Digital SERS Profiling of Structurally Dynamic Antigens via a Multi‐Epitope Bispecific Antibody Framework
Source: Adv Sci (Weinh). 2026 May 27;13(46):e75828. doi: 10.1002/advs.75828 (PMC13336131; doi:10.1002/advs.75828)
Supplement: Supplementary file 1 [file ADVS-13-e75828-s001.docx]

**Supporting Information**

**Epitope-Resolved Digital SERS Profiling of Structurally Dynamic Antigens via a Multi-Epitope Bispecific Antibody Framework**

Jing Wang^a,b,^*, Quan Zhou^b^, Kym Lowry^c,d^, Christopher B. Howard^b,^*, Matt Trau^b,e,^*

^a^Key Laboratory of OptoElectronic Science and Technology for Medicine of Ministry of Education, Fujian Provincial Key Laboratory of Photonics Technology, Fujian Normal University, Fuzhou 350117, China.

^b^Australian Institute for Bioengineering and Nanotechnology (AIBN), The University of Queensland, Brisbane, QLD 4072, Australia.

^c^Frazer Institute, Faculty of Health, Medicine, and Behavioural Sciences, The University of Queensland, Royal Brisbane and Women’s Hospital, Brisbane, QLD 4006, Australia.

^d^Queensland Paediatric Infectious Diseases (QPID) Sakzewski Laboratory, Queensland Children’s Hospital, Brisbane, QLD 4101, Australia.

^e^School of Chemistry and Molecular Biosciences, The University of Queensland, Brisbane, QLD 4072, Australia.

*Corresponding authors: jing.wang@fjnu.edu.cn; c.howard2@uq.edu.au; m.trau@uq.edu.au

Figure S1. SDS-PAGE gel staining of α21, α34, and α105.

Figure S2. Determination of the enhancement factor of gold–silver nanoboxes (NBs). (a) Concentration-dependent SERS spectra of MBA (0.01–0.1 µM) functionalized on NB surfaces. (b) Conventional Raman spectra of MBA in solution at concentrations ranging from 10 mM to 100 mM. The enhancement factor was calculated as the ratio of the slopes of the linear calibration curves obtained from SERS (b) and conventional Raman measurements (a)^1^.

Figure S3. (a) DCS and (b) Raman spectroscopy measurements of SERS nanotags (NB-MBA) conjugated with varying amounts of 1 mM mPEG.

Figure S4. (a) Stepwise SERS spectra of a representative nanotag during surface modification, together with (b) the final spectra of all three epitope-specific nanotags used for multiplexed detection. For (a), the nanotags were prepared by co-functionalizing NBs with the Raman reporter (MBA) and mPEG, followed by BsAb (α21) conjugation.

Figure S5. Nanoflow cytometry of BsAb-functionalized SERS nanotags in the presence of GFP-tagged RBD and 1% (w/v) BSA. SS-H: side scatter height; GFP-H: green fluorescence height. Inset values indicate the percentage of BsAb-functionalized nanotags emitting GFP signals.

Figure S6. DCS analysis of immunocomplexes formed between (a) α21/α34- and (b) α21/α105-modified SERS nanotags and RBD at inputs from 0 to 500 ng.

Figure S7. SERS detection RBD captured by CR3022 IgG subclasses and probed with BsAb-modified SERS nanotags. Comparison of CR3022 mAb stored at 4 °C for 7 days (Day 7) versus freshly thawed (Day 0). *P<0.0332, *P<0.0021, *P<0.0001. Statistical significance was assessed using the two-way ANOVA.

Figure S8. Single-epitope ROC analyses. Receiver operating characteristic (ROC) curves for individual epitope channels based on digital active-pixel counts obtained from clinical samples using (a) α21-, (b) α34-, and (c) α105-functionalized SERS nanotags. The corresponding areas under the curve (AUC) are indicated in each panel.

Figure S9. Performance of the multi-epitope multiple logistic regression model evaluated by 5-fold cross-validation. (a) ROC curve and (b) confusion matrix at a threshold of 0.5.

**Reference**

1.	Yue, X.; Yan, S.; Gao, T.; Pu, S.; Tang, H.; Pei, X.; Tian, Z.; Wang, X.; Ren, B.; Liu, G., SERS Performance Factor: A Convenient Parameter for the Enhancement Evaluation of SERS Substrates. *Anal. Chem.* **2024,** *96* (44), 17517-17525.
